# Eco-friendly synthesis of chitosan and its medical application: from chitin extraction to nanoparticle preparation

**DOI:** 10.5599/admet.1999

**Published:** 2023-09-23

**Authors:** Riyona Desvy Pratiwi, Sjaikhurrizal El Muttaqien, Nunik Gustini, Najla Salsabilla Difa, Gita Syahputra, A’liyatur Rosyidah

**Affiliations:** Research Center for Vaccine and Drug, Organization Research of Health, The National Research and Innovation Agency, Jalan Raya Bogor Km 46 Cibinong, Bogor 16911, West Java, Indonesia

**Keywords:** green synthesis, nano-chitosan, drug delivery

## Abstract

**Background and Purpose:**

Chitosan, a chitin deacetylation product, has been applied in nanoparticle or nano-chitosan for medical applications. However, the chitin extraction from crustacean shells and other natural resources, chitin deacetylation, and crosslinking of the chitosan forming the nano-chitosan mostly involve hazardous chemical and physical processes. The risks of these processes to human health and the environment attract the attention of scientists to develop safer and greener techniques. This review aims to describe the progress of harmless chitosan synthesis.

**Experimental Approach:**

All strongly related publications to each section, which were found on scientific search engines (Google Scholar, Scopus, and Pubmed), were studied, selected, and then used as references in writing this review. No limitation for the publication year was applied. The publications were searched from April 2022 - June 2023.

**Key Results:**

Nano-chitosan could be synthesized in harmless techniques, including the preparation of the chitosan raw materials and crosslinking the chitosan polymer. Enzymatic processes in shell deproteination in the chitin extraction and deacetylation are preferable to reduce the negative effects of conventional chemical-physical processes. Mild alkalines and deep eutectic solvents also provide similar benefits. In the nano-chitosan synthesis, naturally derived compounds (carrageenan, genipin, and valinin) show potency as safer crosslinkers, besides tripolyphosphate, the most common safe crosslinker.

**Conclusion:**

A list of eco-friendly and safer processes in the synthesis of nano-chitosan has been reported in recent years. These findings are suggested for the nano-chitosan synthesis on an industrial scale in the near future.

## Introduction

Nanotechnology is a developing field of research for designing tools and systems with specific cellular, atomic, and molecular features involving nanomaterials of size 1-100 nm. Nowadays, the application of nanotechnology has been expanded in the field of biomedical research and clinical practice called nanomedicine [1]. Nanomaterials are used in imaging, drug delivery, and diagnostics in nanomedicine. Since they are small, have a high surface area, and are polydisperse, nanomaterials may have different properties from bulk materials. One of the main advantages of nanoparticles is their small size, which enables them to circulate within the body without obstructing blood flow and to avoid being cleared by the complement and renal systems [2].

Chitosan is a naturally occurring polysaccharide primarily made up of (1-4)-linked glucosamine units with a smidgen of N-acetylglucosamine units. Chitosan is a promising material for medicine and pharmacy due to its biocompatibility—biodegradability, low toxicity, and mucoadhesiveness [3]. Nano-chitosan exhibits various applications in medicine and health. The system is suitable for mucosal delivery, such as oral application, nasal drug, pulmonary, muco-adhesin, and other mucosal routes [4]. Like other largely polymeric nanoparticles, nano-chitosan is biodegradable, biocompatible, and applicable for a broad range of active pharmaceutical ingredients, either small or large molecules, for instance, proteins. The nano-sized chitosan offers numerous valuable chemical, physical, and biological properties for better therapy outcomes [5].

In terms of environmental issues, experts agreed that the final product of nanomedicine, such as nano-chitosan, is not necessarily considered to contribute to high ecological risk [6]. However, from past experiences, chitosan was prepared through a rough process, including irritative chemicals in high concentrations [7]. Moreover, in the earlier years, the chitosan particles were synthesized by threatening substances, for instance, glutaraldehyde [8]. This article aims to review a list of eco-friendly processes to prepare the nano-chitosan, from the extraction of chitin as the main raw material, chitosan synthesis by the chitin deacetylation, and selecting the greener and safer crosslinkers in order to form the nano-chitosan.

## Conventional method for chitin and chitosan production

**Figure 1.**
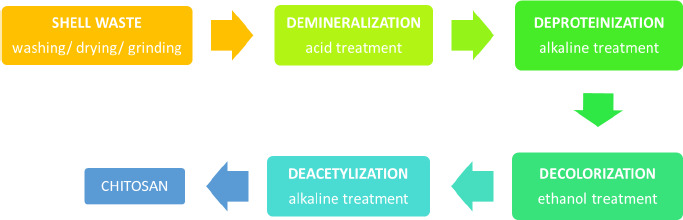
Reaction step of conventional chitosan production

Chitin is an abundant polysaccharide in nature after cellulose in the fungal cell wall and exoskeleton of insects, arthropods, crustaceans, and other invertebrates [9,10]. It has been discovered and explored in several periods since the late eighteenth century [10]. The chemical structure of chitin and cellulose is similar, yet hydroxyl at cellulose C-2 is substituted with an acetamide group in chitin [11]. Chitin is a polymorphic compound with α, β, and γ configurations [12]. It is obtained by extraction from those natural sources, but mostly from shrimp and crab, as up to 80 % of these animals are food wastes [13]. In brief, there are several steps of industrial production of chitin: demineralization, deproteination, bleaching/discoloration, and deacetylation to obtain chitosan, as illustrated in Figure 1. The lengthy extraction process usually takes 17 to 72 h, which includes 1 to 24 h of treatment with hydrochloric acid and 16 to 48 h of sodium hydroxide processing, resulting in a high cost, high energy, and non-environmentally friendly process.

The synthesis is started by grinding the shrimp waste into smaller sizes, followed by the removal of the contained mineral, including calcium carbonate, calcite, and calcium phosphate (demineralization). In this demineralization step, excessive treatment using dilute acidic solutions such as sulfuric, hydrochloric, nitric, acetic, oxalic and formic acids is done, in which the use of hydrochloric acid during the extraction is preferable to obtain a better quality of chitin [14]. After stirring the pulverized dried shells in acidic solution at room temperature, the mineral such as calcium carbonate is transformed into precipitated calcium chloride and carbon dioxide. Following the demineralization step, the reaction mixture should be rinsed with distilled water to neutralize the pH of the mixture. As chitin occurs naturally in association with protein (chitinprotein), a deproteinization process is needed to disrupt the chemical bonds between chitin and proteins. It is done by treating it with aqueous solution of sodium hydroxide (1-10 %) at elevated temperature (65 to 100 °C) for 0.5 to 12 h. In this step, complete removal of the protein from chitin is preferred, particularly for the biomedical application of chitosan, as the remaining shellfish protein may induce allergic reactions in humans. These acid and alkaline treatments resulted in coloured chitin products. The next step is decolorization to remove the red or orange pigment-formed complexes with chitin. The process could be accomplished mainly by treating it with ethanol or acetone and bleaching it with sodium hypochlorite, yielding a white-coloured chitin powder.

To convert chitin to chitosan, the resulting chitin should be deacetylated in the concentrated sodium or potassium hydroxide solution [15]. The acetyl group in the chitin structure causes low solubility and reactivity in many solvents; thus, it is not convenient to use [9]. In 1859, C. Rouget found that the solubilization of chitin in acid increased after being reacted with concentrated potassium hydroxide at high temperatures [16]. This process yields a derivate compound with better solubility and reactivity than its native substance because of the presence of primary and secondary hydroxyl groups [16-18]. The deacetylated chitin was then identified as chitosan by Felix Hoppe Seyler in 1894 [16,19]. After deacetylation, the typical characteristic of each chitin polymorph disappears and becomes amorphous chitosan [12].

**Figure 2.**
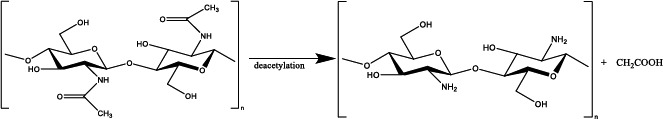
Chitosan as a deacetylation product of chitin

Chitosan itself is a cationic polymer (one positive charge for each glucosamine residue) derived from partial (at minimum 50 %) or almost complete alkaline deacetylation (reaching 95 %) at C-2 chitin which presents -NH_2_ primary group (Figure 2) [20,21]. Among many chitosan derivates such as glycosamine, tosyl - and trityl chitin, chitosan is the most widely used in biomedical and nutraceutical fields [22]. Consisting of amine moiety, chitosan has beneficial biological properties such as biodegradable, highly biocompatible, non-toxic, and low allergenic [23]. Moreover, because of the amino groups, chitosan is a weak base with p*K*_a_ 6.3 to 6.5, which is protonated below physiological pH, leading to application in a pH-responsive system [24]. Because of the D-glucosamine, chitosan is generally insoluble in water and organic solvents and and is soluble in acidic aqueous solutions, such as acetic acid. However, the solubility highly depends on the degree of deacetylation (DD) [24].

DD is a parameter to determine the chitosan characteristic and its biological activity. For instance, aqueous soluble chitosan requires at least 50 % DD, while enzyme or lysozyme non-degradable chitosan must be 95 % DD [25,26]. This parameter is calculated as a unit ratio of 2-acetamido-2-deoxy-glucopyranose (GIcNAc) to 2-amino-2-deoxy-D-glucopyranose (GIcN)) which are linked by a β (1-4) glycosidic bond [27]. DD of the chitosan is analysed by various methods, such as hydrogen bromide titrimetry, infrared spectrometry, UV-Vis spectrophotometry, and ^1^HNMR spectrometry [28-31].

Besides the excessive need for acid and basic chemicals for chitosan production, which affects the quality of the obtained biopolymer, the waste generated from this well-established process should also be carefully considered. The wastewater containing diluted proteins and huge amounts of chloride, calcium, and sodium could potentially induce marine and freshwater ecotoxicity as well as human carcinogenic toxicity [32,33]. Numerous proteins, peptides, and chitooligosaccharides were produced during the deproteinization step as impurities could not be utilized further due to the high level of alkali solvents contained [34]. Neutralization and detoxification of the discharged wastewater using large amounts of fresh water are necessary to avoid waste disposal problems and reduce the environmental impact, leading to the increased cost of the chitin-purification process. Therefore, more eco-friendly processes of chitosan production are needed as alternative methods.

## Eco-friendly chitin and chitosan production

The eco-friendly method for chitosan production was introduced to resolve the shortcomings of the conventional counterparts. The green extraction technique could be done through numerous approaches, such as microbial fermentation, enzyme-assisted extraction, and microwave-assisted extraction. For example, the use of organic acid and protease-producing microorganisms like lactic acid bacteria could replace those chemicals in demineralization, deproteinization, and deacetylation steps without critically affecting the yields of chitin and chitosan [7,35,36]. The lists of enzymatic-producing bacteria used in the chitosan production method have been comprehensively reviewed [37]. In the demineralization step, the use of organic lactic acid and/or acetic acid could result in a relatively comparable effectivity to that obtained with hydrochloric acid [38]. Another benefit of using this biological extraction approach is that the obtained protein-rich by-product could be utilized for other applications, including feed or fertilizer, due to the absence of corrosive chemicals. The resulting wastes from this approach are less harmful to the environment while preserving the characteristics of the obtained chitin. Also, a strong base for neutralization is not needed in the demineralization step using organic acid, limiting the production of salt that could inhibit the enzymatic activity during the enzyme-based deproteinization process. Such a combination of greener routes of organic acid demineralization using citric acid and enzymatic deproteinization using papain or bromelain is beneficial and was reported recently by Pérez to produce chitosan from shrimp shells [39].

In the enzyme-assisted extraction approach, Younes and coworkers [40] used a non-commercial *Bacillus mojavensis* A21 crude enzyme for the deproteinization step of shrimp shells. They optimized several operating parameters, such as enzyme/substrate ratio, temperature and incubation time, by response surface methodology (RSM). The optimal conditions were an enzyme/substrate ratio of 7.75 U/mg, a temperature of 60 °C, and an incubation time of 6 h for obtaining a deproteinization degree of 88 ± 5 %. Valdez-Peña *et al.* explored the potency of commercial enzymes (Alcalase®, Flavorzyme®, Lysozyme, Inovapure 300, Trypsin VI, Papain DSM) on the deproteinization step of shell-shrimp powder [41]. From this research, a high weight loss of 61 % was achieved by using Alcalase®, while using other commercial enzymes resulted in 35 to 38 % weight losses. The use of *Streptomyces griseus* protease for enzymatic protein removal from a shrimp-shell powder of *Litopenaeus vannamei* was reported by Hongkulsup and coworkers [42]. This process achieved 91 % deproteinization at 37 °C, 3 h and 1 wt.% of the enzyme.

In the deacetylation step, the combination of the enzymatic method with a mild alkaline solution was advantageous for the quality of chitin and ecological compensation [43]. In the earlier, chitin deacetylation was only performed using highly concentrated sodium hydroxide reaching 60 % at high temperatures (65 to >100 °C) and long incubation time [44]. Different times, temperatures, and concentrations of the alkaline solution affect the number of DD and molecular weight [45,46]. Higher temperature and hydroxyl solution concentration resulted in higher DD, while after saturation, prolonging the incubation time did not significantly increase DD [47]. High DD (>90 %) was only reached in high hydroxyl solution at high temperature and long incubation. On the contrary, the extreme deacetylation condition damaged the polysaccharide chain, yielding low molecular weight (MW) chitosan [48]. According to the MW, chitosan is classified into three groups, *i.e.* low MW (<100 kDa); medium MW (100 to 1,000 kDa), and high MW (>1,000 kDa) [49]. High DD and low MW chitosan show antimicrobial and antioxidant activity, high DD and high MW chitosan are needed for mucoadhesive activity and application in regenerative medicine or drug delivery, meanwhile, medium MW chitosan has anticancer activity [50,51]. In recent years, modified chitin deacetylations were proposed in order to obtain high DD with lower side effects on the polysaccharide chain. As mentioned above, chemical chitin deacetylation, particularly on a manufacturing scale, inflicts environmental pollution [52]. It was reported that to produce one kilogram of 70 % deacetylated chitosan, at least six kilograms of hydroxy chloride, two kilograms of sodium hydroxide, and a large quantity of nitrogen, water, and organic solvents are customized [53]. Thus, biological processes involving deacetylase enzymes are preferable [54].

The first reported chitin deacetylase (CDA) was identified and purified from *Mucor rouxii*, a dimorphism (yeast-like and filamentous) fungi microbial. The enzyme is a 75 to 80 kDa glycoprotein with high mannose, approximately 30 % carbohydrate, and commonly expressed in the periplasmic space but also secreted to the extracellular medium [55,56]. The enzyme activity was optimum at pH 4.5, 50 °C for glycol chitin model substrate and catalysed at least four N-acetylglucosamine [56]. Afterwards, numerous CADs from other fungi were discovered, isolated, and studied on chitosan production [57]. The CADs have typical biochemical properties, including different molecular sizes from 12 to 150 kDa, acidic pI (2.7 to 4.8), and show optimum bioactivity at 30 to 60 °C in pH 4.5 to 12 [58]. The inefficient yield of the isolated CADs initiated the production of recombinant CADs [59]. Hitherto, several recombinant CADs genes from *Colletotrichum lindemuthianum*, *Saccharomyces cerevisiae*, and *Mucor circinelloides* have been expressed in different bacterial or yeast expression systems such as *Escherichia coli* or *Pichia pastoris*, respectively, and showed proper biological activity [59-65].

As microwave has a high potential to accelerate chemical reactions and increase the reaction yield, a chitin deacetylation method using microwave energy was also introduced to reduce incubation time in the hydroxyl solution from hours to less than twenty minutes [66]. The microwave exposure reduced the heating temperature as well as shortened the heating time due to its heating efficiency and homogeneous microwave field within the sample, generating uniform heating [67,68]. Also, the obtained chitosan products from chemical and microwave energy-assisted extraction displayed similar structures, morphologies and chemical compositions. The direct and quick energy transfer of the microwave irradiation to the substrate and catalyst may be responsible for increased reaction efficiency [69]. In this approach, various process parameters, including reaction time, solvent concentration, and solid-to-liquid ratio, should be optimized to obtain the designated degree of deacetylation and molecular mass of the obtained chitosan.

In addition, the concentration of hydroxyl solution could be decreased by combination with urea [70]. The deep eutectic solvents (DESs) approach proved more sustainable, biocompatible, inexpensive, and more convenient in preparation [71]. DESs are a mixture of at least one hydrogen-bond acceptor (HBA), such as halide salts, and one hydrogen-bond donor (HBD): urea, glycerol, benzamide, and citric acid [72]. The application of the DESs in chitosan production was not only effective in the deacetylation process but also in the chitin extraction, including deproteination and demineralization [73].

Aside from deacetylation products, natural chitosan has also been found in some micro and invertebrate organisms. From *M. rouxii*, the first natural chitosan was isolated from the cell wall using an acidic solution, hydrochloric acid, and formic acid [74]. Afterwards, the natural chitosan was also discovered from other microorganisms like bacteria (*Serratia sp*. and *Bacillus sp*.), yeast (*Candida albicans* and *S. cerevisiae*), and other fungal microorganisms (*Rhizopus oryzae*, *Gongronella butleri*, *Cunninghamella elegans*, and *Phycomyces blakesleeanus* [53]. Zhang and colleagues reported that natural chitosan was only found in budding spores of *S. cerevisiae*, not in the vegetative form [75]. Chitosan extracted from *S. cerevisiae* and *Aspergillus niger* had almost similar DD with chitosan processed from chitin’s shrimp [76]. Producing natural chitosan from those sources reduces chemical and toxic materials during the process, including chitin extraction and deacetylation [53].

Considering the risk and benefit of each process, chemically processed chitin-derivate chitosan is still widely produced on the industrial scale. The enzymatic deacetylation encompasses an eco-friendly method; even in economic value, the enzyme is relatively higher priced than the chemicals [57]. Likewise, the natural chitosan final yield is still relatively low compared with the chitin-derivate chitosan and highly depends on the culture medium and incubation condition [53,76]. Modified chemically processed chitosan has become interesting because of the rational cost and benefit of reducing harm by the conventional chemical method [57].

## Applications of chitosan in nanomedicine

Chitosan has been used and reported in many fields, for instance, in the food and beverage industry; pharmacy and health, including biotechnology, biomedicine, dentistry, and veterinary; agriculture and aquaculture; textile; pulp and paper industry; chemistry and environmental chemistry [77]. In pharmaceutical and health, chitosan has been applied in drug and gene delivery systems, tissue engineering and artificial implants, bioimaging, protein binding, wound healing, and contact lenses [78].

As the concept of nanotechnology introduced by Richard Feynman in 1959, the application of nanotechnology in medicine has been also emerged [79,80]. Nanotechnology in medicine, terminologically defined as nanomedicine, is nano-scaled medicine obtained from an engineering process resulting in different physical, chemical, and biological features than its original material for diagnosis, monitoring, control, prevention, and treatment of diseases [81,82]. However, the exact definition of nanomedicine is still debated among scientists, regulatory agencies, and enterprisers related to size, organic/inorganic material, pharmacodynamic profile, pathology, and route of administration [83]. In general, nanomaterial should be between 1 to 100 nm in size. Still, for nanomedicine, the material, particularly organic nanomedicine such as liposome or micelle, which is more than 100 nm (*i.e.*, 400 nm), is also considered as ‘nano,’ as long it shows significantly improved properties (*i.e.*, higher solubility and bioavailability) than the bulk material [83]. On the other hand, iron oxide only exhibits altered characteristics below 20 nm [83]. In another literature, nanomedicine is determined in the 1 to 1000 nm range [84], but in the context of pathology and route of administration (*i.e.*, enhanced permeability and retention for cytotoxic therapy), the effect could not be observed from >200 nm particles [85].

According to the types of material, nanomedicine is classified into seven groups: oxide/metal nanoparticles; polymer-based nanoparticles; lipid-based nanoparticles; micelles, liposomes, protein-based nanoparticles, and unspecified others [86,87]. Chitosan-based nanoparticle or nano-chitosan is categorized in polymer-based nanoparticles [88]. In the field of medicine, nano-chitosan has been explored for small and large molecule delivery carrier, wound healing, and tissue regeneration, besides the potential as antimicrobial/anticancer/antioxidant given from the chitosan itself [88].

## Eco-friendly crosslinkers for nano-chitosan preparations

Nano-chitosan (and micro-chitosan) is formed by several methods, *i.e.*, ionic gelation crosslinking, reverse micelles, emulsification, precipitation, radical polymerization, and top-down method [8]. The nano-chitosan is characterized differently by each method [89]. Emulsification, reverse micelles, and precipitation involve aqueous chitosan, stabilizer, and organic phases. On the other hand, the organic solvent-free method is much preferred to those methods in which the usage of organic solvent is required. Thus, to date, ionic gelation is the most favourable method for nano-chitosan production. The particle is produced by ionic interaction between positively charged amine groups of chitosan with polyanions crosslinker [8,89].

**Figure 3.**
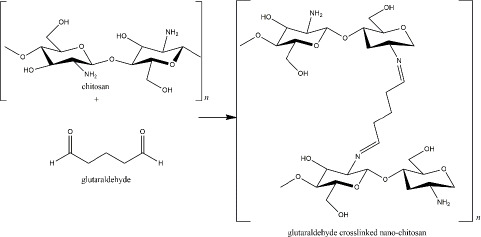
Glutaraldehyde crosslinked nano-chitosan

At the beginning of chitosan development, glutaraldehyde was used as the crosslinker [8]. The crosslinking is formed via imine bonds by the interaction of amine groups of the chitosan and an aldehydic group of the glutaraldehyde. The amount of glutaraldehyde affects the particle size as the higher concentration of glutaraldehyde reduces particle size [90]. In addition, the reaction of glutaraldehyde-chitosan depends on the pH solution, which determines the protonation of the amine groups (Figure 3) [91]. Glutaraldehyde is known as a stable crosslinker for chitosan. However, because of its high toxicity to human health, for instance, skin and eye irritation, respiratory tract problems, and environmental issues, it has been substituted with other safer crosslinkers, such as tripolyphosphate (TPP) [92,93]. As described in Table 1, TPP has been the most commonly used crosslinker for nano-chitosan production. Besides the TPP, some crosslinkers, which are natural-derived compounds, have been reported to exhibit remarkable advantages as harmless crosslinker in the nano-chitosan synthesis (Table 2).

**Figure 4.**
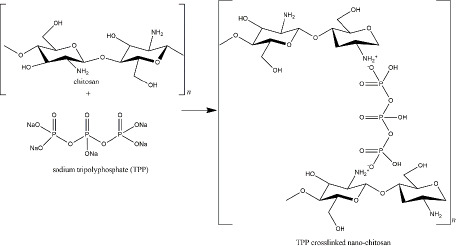
TPP crosslinked nano-chitosan

TPP, also available in sodium TPP (Na_5_P_3_O_10_), reacts with the amine groups of the chitosan through the negatively charged P_3_O_10_^5-^ (Figure 4). Characteristics of nano-chitosan from the chitosan polymer and TPP depend on several experimental factors, such as the ratio of chitosan and TPP, pH solution, ionic strength, stirring type, and rate [131]. TPP was reviewed as the smallest and strongest nano-chitosan’s crosslinker among the others crosslinkers [132]. To overcome the environmental issues, a certain amount of innovative natural-based crosslinker for nano-chitosan has been explored, including carrageenan, genipin, citric acid, and vanillin [129].

Carrageenan is a sulphated galactosan extracted from the *Rhodophyceae* family containing main sugars (o-galactose) and 3,6-anhydro-o-galactose; the main substituent (sulphate); carbohydrate residues (xylose, glucose, and uronic acids), and other substituents (methyl esters) [141]. It is an anionic polymer because of the high component of the ester-sulphate. In addition, because of variations in the sulphate content, carrageenan is classified into six types (κ, λ, ι, θ, β, ν, and μ carrageenan). Each type shows different physiochemical properties (such as solubility and gelling mechanism) and bioactivity [142].

**Figure 5.**
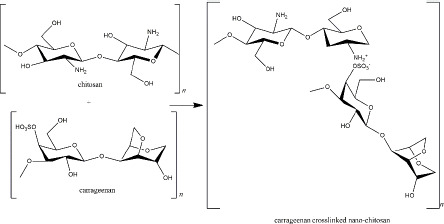
Carrageenan crosslinked nano-chitosan

As a negatively charged polymer, carrageenan is suitable as a crosslinker in the nano-chitosan preparation. Carrageenan (κ-carrageenan) and chitosan were used to form polyelectrolyte complexes (PEC) (Figure 5). Not only does it function as a drug delivery system, tissue regeneration, cell cultivation, and enzyme immobilization, but the PEC also exhibits biological activity as an anti-inflammatory via histamine activation [143].

Khaliq and colleagues reported that cefotaxime sodium loaded κ-carrageenan - chitosan hydrogel was applicable to dress diabetic wounds by releasing the drug in 24 h for effectively inhibiting bacterial propagation [144]. The drug release profile depends on p*K*_a_ or pI and the extrinsic pH. For instance, diflunisal (p*K*_a_ = 2.94) was released from a κ-carrageenan - chitosan nanocapsule in intestinal fluid and blood distribution (pH 7.4) in which the drug was found in anionic and highly soluble form. Otherwise, in lower pH, such as in acidic gastric fluid (pH 2-3), diflunisal was neutral and insoluble in an aqueous solution [145]. Along with TPP, the combination of these two marine polymers was also used for the delivery system of α-mangostin and showed cytotoxicity in the MCF-7 cell line and antibacterial activity [146,147].

**Figure 6.**
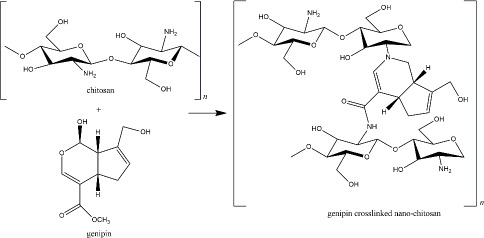
Genipin crosslinked nano-chitosan

Genipin, another naturally derived crosslinker in nano-chitosan preparation, is found in *Genipa americana* fruit. It was soluble in aqueous solution, alcohol, and propylene glycol, less toxic than other synthetic crosslinkers (showed 0.01 % cytotoxicity compared with glutaraldehyde), and reported for several pharmacological activities [148]. This compound actively reacts with primary amine groups of chitosan by substituting oxygen at the dihydropyran ring and the aldehyde group, which depends on environmental pH and oxidation level (Figure 6) [149,150]. Most of the recent applications of genipin-crosslinker chitosan were proposed for tissue regeneration and wound healing. Heimbuck and team described the capabilities of genipin-crosslinked chitosan hydrogel to hamper bacterial growth and to neutralize environmental pH as a wound dressing [151]. A list of genipin-crosslinked chitosan for skeletal regeneration has been reviewed by Wang *et al.* [152].

Another fascinating natural chitosan crosslinker is vanillin or 4-hydroxy-3-methoxy benzaldehyde, an aromatic compound extracted from *Vanilla sp*. beans and commonly used in food and beverage industries. The exorbitant cost of the natural vanilla extraction and harvest limitation caused the idea to chemically synthesize the vanillin compound for a more rational commercial value [153]. 4-hydroxybenzaldehyde was used to produce this artificial vanillin.

**Figure 7.**
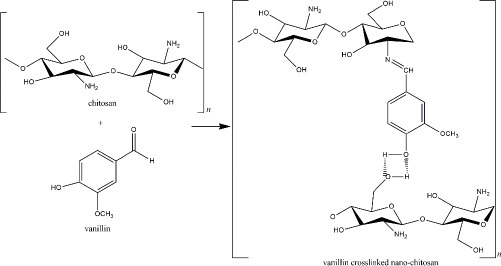
Vanillin crosslinked nano-chitosan

However, the precursor was then reported to harm the environment and human health [153,154]. Therefore, biotechnological-derived approaches, such as plant-based, enzyme-based, and microorganism-based methods, have been applied in vanillin production to obtain lower costs with decreased side effects [153]. The aldehyde group of the vanillin reacts with the chitosan’s primary amine, yielding an imine group (Schiff base). The reaction is reversible by the presence of a para-hydroxyl group (of the imine group) that forms a hydrogen bond with the adjacent hydroxyl of the amino group (Figure 7). Because the reversible hydrogen bond exists, the interaction of vanillin and chitosan relies on pH and temperature. The hydrogen bonds showed high stability at low temperatures [155].

## Discussion and conclusion

The idea of an eco-friendly process, previously called green chemistry, appeared in 1991 to avoid excessive exposure to harmful chemicals to the environment and human health. The harmful chemicals are classified into three following groups: physical problem (explosive and flammable substances); toxicity to human health (causing cytotoxicity, mortality); environmental danger (causing climate change; ozone layer depletion; over-consumption of energy; or uncontrollable waste) [156]. Pharmaceutical industries were reported to contribute significantly to pollutant and waste by-products among chemistry industries. In the last decade, the pharmaceutical industries have applied green chemistry principles as reviewed by Mishra *et al.*, including reducing waste and preventing pollution, using safer chemical or renewable materials, and increasing energy efficiency [157].

Production of nanomaterial for nanomedicine applications, such as nano-chitosan, is also known to involve hazardous substances and waste for the environment and health. Therefore, eco-friendly or green synthesis is requested not only during the nanoparticle assembly but must be applied during the raw materials preparations. In short, two crucial eco-friendly concepts in the nano-chitosan preparation, which are using safer or reducing dangerous chemicals in the chitosan synthesis (including chitin extraction and deacetylation) and determining harmless crosslinkers for the nano-chitosan preparation, have been discussed. Biotechnology processes, particularly enzymatic and microbiological methods, have also been applied in this field, even so, up to now, in terms of industrial capacity and cost, it gives less benefit than the other green methods, for instance, by using sustainable reagents such as DESs. Above all, the natural properties of chitin, chitosan, or nano-chitosan (hydrophilicity, biocompatibility, no organic solvent needed) are the main key to the appropriateness of the eco-friendly or green synthesis concept [158].

## Figures and Tables

**Table 1.** Application of nano-chitosan in medicine

Type	Characteristic	Ref.
Therapeutic protein/peptide carrier	Salivary protein (histatins) delivery, tripolyphosphate (TPP)-crosslinked chitosan	[94]
	Oral delivery of insulin, 3-APBA & L-valine crosslinked carboxymethyl chitosan	[95]
	Oral delivery of insulin, chitosan with SAR6EW (a cell-penetrating peptide)	[96]
	Buccal delivery of bioactive peptide, TPP-crosslinked chitosan	[97]
	Intranasal delivery of recombinant interleukin-17 receptor for asthma, TPP-crosslinked chitosan	[98]
	Delivery of recombinant human bone morphogenetic protein-2, alginate crosslinked chitosan	[99]
Small molecule drug carrier	Oral delivery of rifampicin, TPP-crosslinked chitosan	[100]
	Oral delivery of ciprofloxacin, cyanocobalamin-crosslinked chitosan	[101]
	Targeted delivery of methotrexate for HeLa cells, folic thiolated chitosan	[102]
	Oral delivery of repaglinide and diltiazem HCl, TPP-crosslinked chitosan	[103]
	Delivery of triclosan and flurbiprofen for periodontitis, chitosan nanogels	[104]
	Brain delivery of rotigotine for Parkinson’s disease, TPP-crosslinked chitosan	[105]
Vaccine drug carrier	Cancer vaccine delivery of whole tumor cell lysate, mannose-alginate coated chitosan	[106]
	Avian infectious bronchitis virus (IBV) delivery, TPP-crosslinked chitosan	[107]
	Hepatitis A vaccine delivery, alginate-coated, sodium sulfate-crosslinked chitosan	[108]
	Recombinant hepatitis B surface antigen delivery, TPP-crosslinked, mannosylated chitosan	[109]
	Curdlan intranasal vaccine delivery, quartenized chitosan	[110]
	Vo protein for meningitis vaccine, polylactic glycolic acid - chitosan combination	[111]
	DNA (pDNA-E7mut) vaccine, TPP-crosslinked chitosan	[112]
	Delivery of vaccine for diarrhea (LSC-chimeric recombinant protein), TPP-crosslinked chitosan	[113]
Chitosan-metal nanoparticle complex	Chitosan-polyethylene oxide (PEO) nanofibers combined with silver and zinc oxide nanoparticles for antibacterial wound healing	[114]
	Chitosan-gold nanoparticles for DNA carrier	[115]
Anti-infective wound healing	HemCon®, FDA approved for hemorrhage in 2003	[116]
	Composite sponge of chitosan and hydroxybutyl chitosan	[117]
	Electrospun chitosan nanofibers	[118]
	Microchannelled alkylated chitosan sponge	[119]
	Chitosan microneedle array, antiangiogenic and antibacterial	[120]
	Chitosan-poly vinyl alcohol patch	[121]
	Chitosan-bentonite nanocomposite	[122]
	Chitosan-glucan-collagen complex with aloevera	[123]
	*Hypericum perforatum* oil incorporated chitosan cryogel	[124]
	Chitosan-iron (III) sulfate-levofloxacin dressing	[125]
Tissue regeneration	Nerve tissue regeneration, chitosan-collagen composite scaffold for Schwann cells	[126]
	Skin tissue regeneration, keratinocyte loaded chitosan-polyvinyl alcohol-silk electrospun system	[127]
	Bone tissue regeneration, gelatin-chitosan scaffold for hydroxyapatite (HAp), β-tricalcium phosphate (β-TCP) and 58s bioactive glass	[128]
	Bone tissue regeneration, composite of chitosan, chondroitin sulfate, and nano-bioglass	[129]
	Cartilage tissue regeneration, chitosan-poly(L-lactide)-peptin composite	[130]

**Table 2.** Eco-friendly crosslinkers in nano-chitosan production

Crosslinker	Application	Materials	Ref
Tripolyphosphate (TPP)	Nanocomposite film of gelatin-coated chitosan-sodium TPP nanoparticles for wound healing	0.5 % chitosan in 2 % acetic acid pH 4.5; 10 mL sodium TPP solution (0.7 mg/mL)	[133]
	Nanocomplex of cashew gum, chitosan, and TPP for insulin loading	0.2% chitosan in 0.1 % lactic acid pH 3.4; 13.5 mM TPP pH 9.1; cashew gum 0.5 % pH 6.2	[134]
Carrageenan	Slow-release nanoparticles/polyelectrolytes complexes of chitosan and κ-carrageenan	100 mg chitosan and 100 mg κ-carrageenan in 0.2 M sodium acetate buffer (pH 3-6)	[135]
	Polyelectrolytes complexes of chitosan and κ-carrageenan for protein delivery	1.5 g chitosan in 400 mL acetic acid 1 %; 1.5 g κ-carrageenan in 100 mL deionized water enriched with 25 g sodium chloride	[136]
Genipin	Biocompatibility study of genipin-chitosan hydrogels for vaccine delivery	1.5 % chitosan in 1 % acetic acid. Ratio chitosan: genipin = 1: 0.1 to 0.3	[137]
	Genipin crosslinked caseinate-chitosan for curcumin delivery as an anticancer drug	1 mg/mL chitosan in 0.1 M acetic acid; 20 mg/mL genipin in DMSO	[138]
Vanillin	Vanillin-chitosan particles for zinc sustained release system	Molar ratio of vanillin: chitosan (0.1 to 2.0) in 3 mL glacial acetic acid; loaded with 1g/L zinc acetate ethanol solution pH 6	[139]
	Cytotoxicity drug-loaded vanillin-chitosan nanoparticles	2 % chitosan in 1 % acetic acid (10 mL); 25 mg/mL vanillin (50 mL); 50 mg 5-fluorouracil	[140]
